# Value of routine heart rate variability parameters for atrial fibrillation detection in ischaemic stroke and high-risk TIA patients

**DOI:** 10.3389/fstro.2025.1727719

**Published:** 2026-01-21

**Authors:** Kurt Moelgg, Anel Karisik, Lucie Buergi, Lukas Scherer, Luisa Delazer, Benjamin Dejakum, Silvia Felicetti, Theresa Koehler, Julian Granna, Christian Boehme, Raimund Pechlaner, Theresa Prock, Thomas Toell, Axel Bauer, Michael Schreinlechner, Daniel Pavluk, Michael Knoflach, Stefan Kiechl, Lukas Mayer-Suess

**Affiliations:** 1 Department of Neurology, Medical University of Innsbruck, Innsbruck, Austria; 2 VASCage, Centre on Clinical Stroke Research, Innsbruck, Austria; 3 Department of Internal Medicine III, Cardiology and Angiology, Medical University of Innsbruck, Innsbruck, Austria

**Keywords:** atrial fibrillation, heart rate variability, holter ECG, ischaemic stroke, risk prediction, transient ischaemic attack

## Abstract

**Introduction:**

Undetected atrial fibrillation (AF) increases the risk of recurrent ischaemic stroke, but current prediction scores do not incorporate heart rate variability (HRV) measures readily available from 24-h Holter ECGs.

**Methods:**

In 697 patients with non-AF ischaemic stroke or non-AF high-risk transient ischaemic attack (TIA) from the STROKE-CARD Registry (NCT04582825), we assessed eight time-domain HRV parameters for predicting incident AF within 1 year. ROC analyses, logistic regression, and the Youden index were used to identify optimal cut-offs and compare HRV performance with Brown-ESUS AF and AS5F scores.

**Results:**

New-onset AF was detected in 28 patients (4.0%). PNN50, rMSSD, and SDSD showed the best discrimination (AUC = 0.711, 0.766, and 0.775), outperforming both clinical scores (AUC ≤ 0.612). Optimal cut-offs were 5.5% (PNN50), 48.5 ms (rMSSD), and 43.5 ms (SDSD). Dichotomized analyses confirmed strong associations with AF (ORs 5.34–7.70, all *p* < 0.001), and adding HRV parameters significantly improved prediction beyond existing scores.

**Conclusions:**

PNN50, rMSSD, and SDSD from routine Holter ECGs enhance AF risk prediction after non-cardioembolic stroke or high-risk TIA and may support targeted monitoring strategies.

## 1 Introduction

Ischaemic stroke is the third leading cause of death globally (Feigin et al., 2021). Recurrence of ischaemic stroke, which occurs in up to 12% of patients within the first year, gravely impacts the life of post-stroke patients (Sacco et al., 1994). A key factor in limiting the recurrence of ischaemic stroke is the clear identification of stroke etiology. Still, the etiology of ischaemic stroke remains unknown in up to 35% of all cases despite comprehensive diagnostic work-up (Kleindorfer et al., 2021). As an example, embolic lesions in ischemic stroke patients remain of unknown source in about 17% (Kleindorfer et al., 2021; Hart et al., 2017). The key issue in this setting is that atrial fibrillation (AF) may initially remain occult, resulting in inadequate secondary prevention, which subsequently entails a higher recurrence risk. AF itself is a major cause of ischaemic stroke, as the arrhythmia promotes thrombus formation in the left atrium, which can embolize and occlude cerebral arteries. Numerous measures have been proposed to improve the detection of occult AF. Risk scores such as the Brown-ESUS AF and the AS5F score, have been developed to identify patients with a high pre-test probability of underlying AF (Ricci et al., 2018; Uphaus et al., 2019). However, these scores do not consider measures of heart rate variability (HRV), a biological marker of autonomic nervous system activity, in the post-stroke setting. HRV itself is a well-established indicator for cardiovascular health, as reduced HRV has been associated with cardiovascular events and mortality (Hillebrand et al., 2013; Orini et al., 2023). Moreover, HRV has been associated with an increased risk of new-onset AF (Orini et al., 2023; Agarwal et al., 2017; Geurts et al., 2023). As these measures are available from routine Holter ECGs, which are considered standard of care in post-stroke work-up, we aimed to investigate the usefulness of individual HRV markers in identifying patients at risk of occult AF in stroke or high-risk transient ischaemic attack (TIA) patients compared to available risk scores. Further, to enhance applicability, it was our goal to establish cut-offs of these values applicable to enable translation into clinical routine.

## 2 Materials and methods

### 2.1 Study population

The STROKE-CARD Registry (clinicaltrials.org identifier NCT04582825) was initiated in December 2020 and is conducted at the Medical University of Innsbruck and Hospital St. John's of God Vienna. The project recruits consecutive patients suffering either acute ischaemic stroke or acute high-risk TIA (ABCD^2^-score ≥4) within 30 days after admission to participating centers and permanent residence in Tyrol (Toell et al., 2018; Willeit et al., 2020). Exclusion criteria were current mandatory military service, imprisonment, or age below 18 years (Toell et al., 2018; Willeit et al., 2020). Patient eligibility is assessed during their hospital stay and written informed consent is obtained from all study participants. Its protocol is based on the STROKE-CARD trial, which established a structured post-stroke disease management program through its positive effect on cardiovascular disease (non-fatal ischaemic stroke, non-fatal haemorrhagic stroke, non-fatal myocardial infarction, or vascular death; Willeit et al., 2020). The STROKE-CARD concept relies on comprehensive clinical as well as laboratory work-up during the initial hospital stay and extends these interventions to in-person follow-up visits 3- and 12-months after index stroke or TIA. This results in individualized in-person post-stroke treatment and personalized risk factor management in consecutive ischaemic stroke and TIA patients. For this current analysis, we examined all non-AF ischaemic stroke or TIA patients, who had Holter (24-h) ECG-derived time-domain HRV parameters recorded during their diagnostic work-up. All individuals included in this analysis had to be in sinus rhythm during Holter ECG recording. The STROKE-CARD Registry has been approved by the Ethics Committee at Medical University of Innsbruck (EK-Nr: 1182/2020) and is being carried out in line with the Declaration of Helsinki. For this secondary analysis, fully pseudonymized patient data were used.

### 2.2 Variable definitions

All HRV parameters were automatically derived from 24-h Holter ECG recordings obtained during inpatient stay, which are routinely performed in our cohort to rule out AF. The eight time-domain parameters derived from Holter ECGs were: (1) SDNN—the standard deviation of NN intervals, (2) SDANN—standard deviation of the average of NN intervals for each 5 min segment of a 24 h HRV recording, (3) SD—Poincaré plot standard deviation perpendicular the line of identity, (4) HRV-TI—total number of all NN intervals divided by the height of the histogram of all NN intervals measured on a discrete scale with bins of 7.8125 (1/128 s), (5) PNN50—integral of the density of the RR intervals that differ by more than 50 ms, (6) rMSSD—root mean square of successive RR interval differences (rMSSD), (7) SDNN-Index—mean of the standard deviation of all the NN intervals for each 5 min segment of a 24 h HRV recording, (8) SDSD—standard deviation of successive differences between adjacent NNs. NN intervals refer to normal-to-normal intervals, which include all intervals between adjacent QRS complexes resulting from a sinus node depolarization (Malik et al., 1996).

Pre-existing conditions, including diabetes mellitus, arterial hypertension, dyslipidaemia, smoking status, and current medication intake are based on detailed patient interview, medical history, and electronic health care records with their definitions adhering to current guidelines. Arterial hypertension was defined according to guidelines as a blood pressure above ≥140/90 mmHg (Williams et al., 2018). Dyslipidaemia at baseline was defined according to the following criteria: total cholesterol ≥200 mg/dl, LDL-C ≥100 mg/dl, HDL-C ≤ 40 mg/dl, TG ≥150 mg/dl, Lp(a) ≥75 nmol/L or intake of lipid lowering therapy. Lp(a) is a genetically determined dyslipidaemia (Reyes-Soffer et al., 2024). According to the European Society of Cardiology (ESC) guidelines, values <75 nmol/L are generally not considered clinically relevant (Kronenberg et al., 2022). Diabetes was considered present if documented in the health record, if the patient was receiving antidiabetic medication, or, in the case of type II diabetes, if HbA1c ≥6.5% (El Sayed et al., 2023). As plasma fasting plasma glucose may be transiently elevated after acute ischaemic stroke due to activation of the hypothalamic-pituitary-adrenal axis, guideline-based definition using blood glucose levels was not applied (Lindsberg and Roine, 2004). Prior cardiovascular events, such as ischaemic stroke, TIA, or myocardial infarction, were identified via patient interview or electronic health records. Severity of ischaemic stroke and TIA at admission was graded by stroke physicians using the National Institute of Health Stroke Scale (NIHSS) and ABCD^2^-Score, respectively (Lyden et al., 2001; Johnston et al., 2007). Stroke etiology was determined by treating stroke experts adhering to the Trial of Org 10172 in Acute Stroke Treatment (TOAST) classification criteria after complete diagnostic work-up (Adams et al., 1993).

### 2.3 Follow-up and primary outcome parameter

Participants were routinely followed-up 3- and 12-months after the index stroke in the STROKE-CARD Registry outpatient clinic. For participants who did not attend the on-site visit, study information was obtained either through standardized telephone assessment or electronic health-care records (ELGA). Our primary outcome parameter was newly diagnosed AF follow-up, based on evaluations by treating cardiologists or specialists for internal medicine. Data were censored at withdrawal from the study, at the end of follow-up, or at death.

### 2.4 Brown-ESUS AF and AS5F score

The Brown-ESUS AF Score has been developed for patient with embolic stroke of undetermined source (ESUS) and is based on age and left atrial enlargement: points are assigned considering patients age (1 point for age 65–74 years, 2 points for age ≥75 years) and left atrial enlargement (2 points if moderate or severe according to the original publication as well as the European Society of Cardiology– diameter ≥43 mm in women and ≥47 mm in men; Ricci et al., 2018; Lang et al., 2015). The AS5F score (range 0–4 points), by contrast relies on age and stroke severity: age in years is multiplied by 0.76, and patients receive 9 points for an NIHSS score ≤ 5 or 21 points for an NIHSS >5 (Uphaus et al., 2019). The strength of both scores is their clinical applicability as they derive from values attained through clinical routine. For this current analysis, values recorded during initial hospital stay for index stroke or TIA were applied to calculate these scores.

### 2.5 ECG pre-processing

ECG signals were analyzed using a light preprocessing strategy to preserve physiological characteristics of ventricular depolarization and repolarization. R peaks were identified using a validated automated QRS detection algorithm and used for beat segmentation and temporal alignment; no manual correction was applied. Raw ECG signals were not subjected to aggressive band-pass or notch filtering to avoid distortion of T-wave morphology. Beat-to-beat repolarization vectors were computed, and the resulting dT° time series was linearly interpolated at 2 Hz and low-pass filtered to suppress isolated artifacts and high-frequency noise. Filtering was applied exclusively to the derived dT° signal and not to the raw ECG.

### 2.6 Statistical analysis

Non-normally distributed numerical variables are analyzed using median and the interquartile range (IQR). Categorical variables were summarized using absolute numbers and percentages. HRV parameters are presented through mean and standard deviation (SD).

Multivariate logistic regression was performed to assess the association between specific linear time-domain HRV parameters and our primary outcomes. Variance Inflation Factor (VIF) was calculated for all covariates included to assess multicollinearity among predictors using a threshold of 3 to identify any significant collinearity concerns.

In subsequent analyses, cut-off values for each HRV time-domain were obtained through receiver operating characteristic (ROC) curves and the Youden's index, which enabled dichotomization. An area under the curve (AUC) between 0.7 and 0.8 was considered acceptable, while an AUC between 0.5 and 0.7 indicated no discrimination (Hosmer Jr et al., 2013).

Covariates used for adjustment of both multivariate logistic regression models were age, sex, admission NIHSS, BMI, smoking status, diabetes mellitus, medication intake like angiotensin-converting enzyme inhibitor, angiotensin receptor inhibitor, beta-blockers, and digitalis. BMI, smoking status, diabetes mellitus, intake of angiotensin-converting enzyme inhibitor, angiotensin receptor inhibitor and beta blockers were selected based on their established associations with new onset of AF reported in the literature (Sha et al., 2024; Chamberlain et al., 2011; Wang et al., 2019; Nair et al., 2014; Habel et al., 2023). To assess the additive predictive value of HRV parameters beyond established clinical scores, we performed hierarchical logistic regression analyses. The respective score (Brown-ESUS AF or AS5F) was entered in the first block, followed by one dichotomized PNN50, rMSSD, or SDSD in the second block. The incremental contribution of each parameter was evaluated using the Omnibus likelihood ratio test, with a significant Δχ^2^ indicating that the HRV parameter provided additive predictive value beyond the clinical score. To evaluate model generalizability and potential overfitting, 10-fold cross-validation was performed for all CHAID models. Statistical analysis was conducted with IBM SPSS Statistics Version 29.0.0.0 (241).

## 3 Results

**Figure 1:**
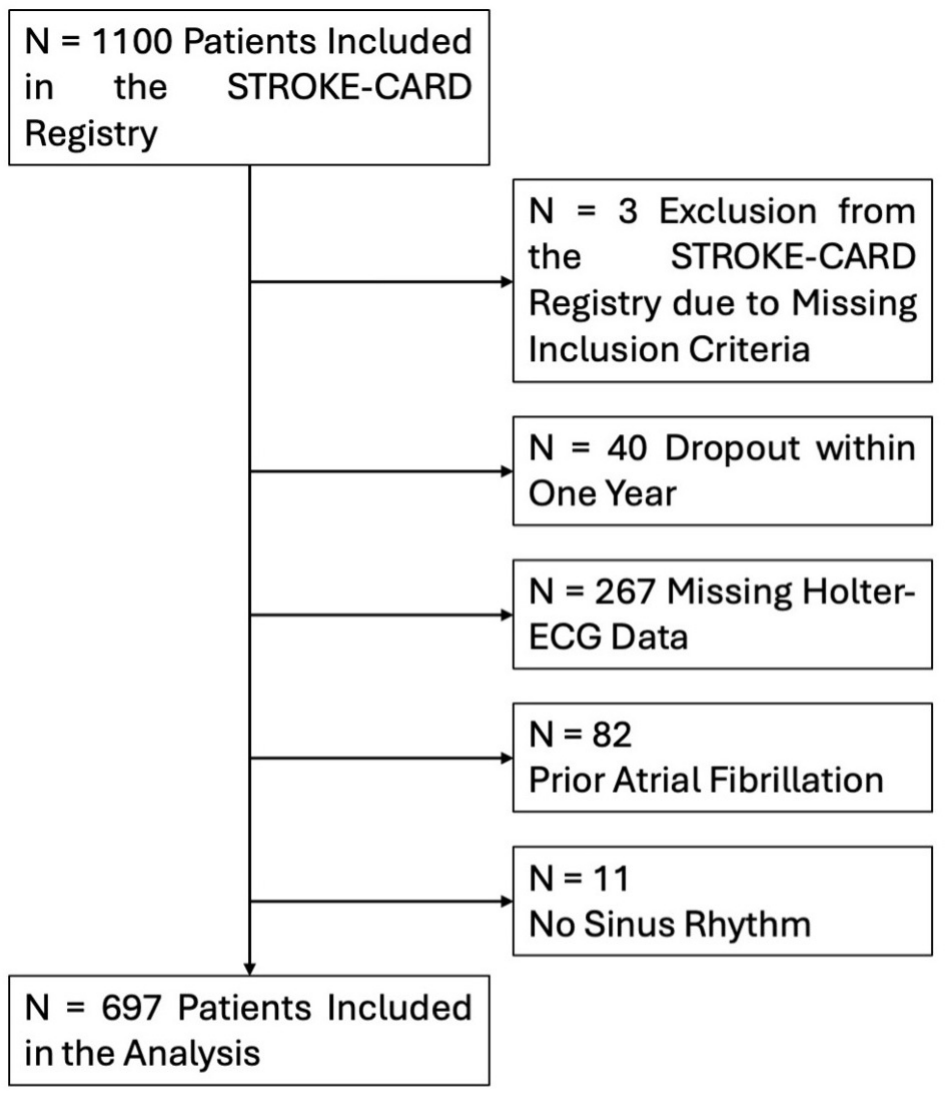
Flow chart presenting the selection of eligible participants.

A total of 1,100 patients were recruited between 09.12.2020 and 23.04.2023. Of these, 697 of 1,100 (63.4%) had ischaemic stroke or high-risk TIA with available Holter ECG data, making them eligible for this analysis (Figure 1). Patient dropout rate was 3.6% of patients dropped out, indicating a low dropout rate. Patients were followed up for a median of 370 days (IQR 365–380). Baseline characteristics, vascular risk factors, and clinical characteristics of the study cohort are presented in Table 1.

**Table 1:** Baseline characteristics of the entire study cohort.

**Characteristics**	**Entire cohort (*n* = 697)**
Age, median (IQR)	71 (61–78)
Female sex, *n* (%)	256 (36.7)
Body mass index, mean (SD)	26.37 (± 4.35)
**Clinical presentation at admission**	
NIHSS, median (IQR)	2 (0–4)
**Lateralization determined by imaging or clinical findings**	
Right hemisphere, *n* (%)	273 (45.5)
Left hemisphere, *n* (%)	317 (39.2)
Both hemipshere, *n* (%)	76 (10.9)
Unknowns, *n* (%)	31 (4.4)
**Cardiovascular risk factors**	
History of hypertension, *n* (%)	428 (61.4)
History of diabetes, *n* (%)	110 (15.8)
History of hypercholesterinaemia, *n* (%)	110 (15.8)
History of ischaemic stroke/TIA, *n* (%)	137 (19.7)
History of myocardial infarction, *n* (%)	78 (11.2)
**Subtypes classified according to the TOAST criteria**	
Large-artery atherosclerosis, *n* (%)	153 (22)
Cardioembolism, *n* (%)	36 (5.2)
Small-artery occlusion, *n* (%)	189 (27.1)
Stroke of other determined etiology, *n* (%)	32 (4.6)
Stroke of undetermined etiology, *n* (%)	287 (41.2)
**Medication**	
ACE-inhibitors, *n* (%)	84 (12.1)
ARB, *n* (%)	259 (37.2)
Beta-blockers, *n* (%)	151 (21.7)
Antiarrhythmic agent, *n* (%)	1 (0.1)

NIHSS, National Institute of Health Stroke Scale; ACE-inhibitors, Angiotensin-converting enzyme inhibitors; ARB, Angiotensin 2 receptor blockers.

During follow-up, 28 of 697 (4.0%) in the entire cohort were diagnosed with AF. Among existing prediction scores, the Brown-ESUS AF score was significantly associated with AF detection [OR 1.41, 95% CI (1.05–1.90), *p* = 0.022], whereas the AS5F score showed a non-significant trend [OR 1.04, 95% CI (0.99–1.08), *p* = 0.058]. Applying metric HRV values in unadjusted logistic regression, a significant association to newly detected AF upon follow-up was only present in SD [OR 1.02, 95% CI (1.00–1.04), *p* = 0.013; Table 2]. However, acceptable AUC of the ROC curve (AUC 0.7–0.8) could be demonstrated for PNN50, rMSSD, and SDSD. Using cut-offs derived from the Youden Index (PNN50: 5.5%, rMSSD: 48.5 ms, SDSD: 43.5 ms) dichotomized unadjusted logistic regression analysis revealed a significant association between all three parameters and newly detected AF upon follow-up: PNN50 [OR 4.62, 95% CI (1.85–11.55), *p* = 0.001], rMSSD [OR 5.95, 95% CI (2.58–13.73), *p* < 0.001], SDSD [OR 6.89, 95% (2.87–16.56), *p* < 0.001]. Results remained robust after adjustment (Table 3).

**Table 2:** Unadjusted analysis of HRV components and incident atrial fibrillation.

**Variable**	**Area under the curve (AUC)**	**Atrial fibrillation, OR^a^ (95%, CI) *p*-value**	**Cut-off**	**Positive predictive value**	**Negative predictive value**	**Sensitivity**	**Specificity**	**Atrial Fibrillation, OR^b^ (95%, CI) *p*-value**
SDNN (ms)	0.576	1.00 (1.00-1.01) *p* = 0.151	≥127.5 ms	6.0%	97.0%	53.6%	64.7%	2.12 (1.00-4.52) *p* = 0.053
SDANN (ms)	0.490	1.00 (0.99–1.01) *p* = 0.556	≥124.5 ms	7.3%	96.8%	35.7%	81.0%	2.37 (1.07–5.26) *p* = 0.034
SD (ms)	0.656	1.02 (1.00–1.04) *p* = 0.013	≥22.5 ms	5.4%	99.5%	96.3%	30.3%	11.32 (1.53–84.01) *p* = 0.018
HRV-TI	0.477	1.01 (0.97–1.06) *p* = 0.546	≥41.5	11.8%	96.5%	14.8%	95.4%	3.63 (1.18–11.16) *p* = 0.024
PNN50	0.711	1.00 (1.00–1.01) *p* = 0.306	≥5.5%	7.0%	98.4%	78.6%	55.8%	4.62 (1.85–11.55) *p* = 0.001
rMSSD (ms)	0.766	1.00 (1.00–1.00) *p* = 0.055	≥48.5 ms	9.2%	98.2%	71.4%	70.4%	5.95 (2.58–13.73) *p* < 0.001
SDNN-index (ms)	0.657	1.01 (1.00–1.02) *p* = 0.064	≥39.5 ms	8.8%	97.4%	50.0%	78.2%	3.58 (1.67–7.68) *p* = 0.001
SDSD (ms)	0.775	1.00 (1.00–1.00) *p* = 0.064	≥43.5 ms	9.9%	98.5%	74.1%	70.7%	6.89 (2.87–16.56) *p* < 0.001

^a^Binary logistic regression using HRV parameters as continuous (linear) predictors. ^b^Binary logistic regression using HRV parameters as dichotomized predictors.

**Table 3:** Adjusted analysis of hrv components and incident atrial fibrillation.

**Variable**	**Area under the curve (AUC)**	**Atrial fibrillation, OR^a^ (95%, CI)** ***p*-value**	**Cut-off**	**Positive predictive value**	**Negative predictive value**	**Sensitivity**	**Specificity**	**Atrial fibrillation, OR^b^ (95%, CI)** ***p*-value**
SDNN (ms)	0.576	1.01 (1.00–1.02) *p* = 0.089	≥127.5 ms	6.0%	97.1%	53.6%	64.7%	2.35 (1.06–5.21) *p* = 0.036
SDANN (ms)	0.490	1.00 (1.00–1.02) *p* = 0.443	≥124.5 ms	7.3%	96.8%	35.7%	81.0%	2.62 (1.12–6.13) *p* = 0.027
SD (ms)	0.656	1.02 (1.00–1.04) *p* = 0.016	≥22.5 ms	5.4%	99.5%	96.3%	30.3%	16.53 (2.15–127.30) *p* = 0.007
HRV-TI	0.477	1.02 (0.97–1.06) *p* = 0.482	≥41.5	11.8%	96.5%	14.8%	95.4%	4.58 (1.35–15.54) *p* = 0.015
PNN50	0.711	1.00 (1.00–1.01) *p* = 0.409	≥5.5%	7.0%	98.4%	78.6%	55.8%	5.34 (2.09–13.66) *p* < 0.001
rMSSD (ms)	0.766	1.00 (1.00–1.01) *p* = 0.103	≥48.5 ms	9.2%	98.2%	71.4%	70.4%	6.61 (2.74–15.96) *p* < 0.001
SDNN-Index (ms)	0.657	1.01 (1.00–1.02) *p* = 0.051	≥39.5 ms	8.8%	97.4%	50.0%	78.2%	4.55 (2.01–10.32) *p* < 0.001
SDSD (ms)	0.775	1.00 (1.00–1.01) *p* = 0.128	≥43.5 ms	9.9%	98.5%	74.1%	70.7%	7.70 (3.06–19.38) *p* < 0.001

^a^Binary logistic regression using HRV parameters as continuous (linear) predictors. ^b^Binary logistic regression using HRV parameters as dichotomized predictors. Both logistic regressions were adjusted for age, sex, smoking status, diabetes mellitus, body-mass-index (BMI), National Institute of Health Stroke Severity Scale (NIHSS), medication intake of angiotensin-converting enzyme inhibitor, angiotensin receptor inhibitor, beta-blockers, and digitalis during inpatient stay.

Additionally, PNN50, rMSSD, and SDSD demonstrated high negative predictive value (98.2%−98.5%) reflecting strong predictive value to rule out the condition in this low prevalence cohort (Tables 2, 3). Among all measures, SDSD and rMSSD provided the most balanced diagnostic performance combining sensitivity and specificity >70% with the highest PPVs (Tables 2, 3).

Importantly, adding dichotomized HRV parameters to existing prediction models significantly improved AF detection. In models including the Brown-ESUS AF score, PNN50 [OR 4.64, 95% CI (1.83–11.74), *p* = 0.001; Δχ^2^ = 12.80, *p* < 0.001], rMSSD [OR 5.49, 95% CI (2.33–12.94), *p* < 0.001; Δχ^2^ = 17.12, *p* < 0.001], and SDSD [OR 6.10, 95% CI (2.47–15.02), *p* < 0.001; Δχ^2^ = 18.03, *p* < 0.001] were all significantly associated with AF detection during follow-up. Comparable results were observed when integrating HRV into models including the AS5F score, with PNN50 [OR 4.54, 95% CI (1.82–11.37), *p* = 0.001; Δχ^2^ = 12.86, *p* < 0.001], rMSSD [OR 5.46, 95% CI (2.33–12.79), *p* < 0.001; Δχ^2^ = 17.27, *p* < 0.001], and SDSD [OR 6.41, 95% CI (2.62–15.70), *p* < 0.001; Δχ^2^ = 19.32, *p* < 0.001] showing similar significant associations. Cross-validation demonstrated that the CHAID models, including those with PNN50, rMSSD, and SDSD, exhibited no evidence of overfitting.

## 4 Discussion

We analyzed consecutive patients with ischemic stroke or high-risk TIA without documented AF to assess the clinical utility of routinely obtained HRV measures in identifying individuals at risk of occult AF. To date, published risk scores have shown fair diagnostic performance for predicting AF detection in patients with cryptogenic stroke, but have not seen widespread clinical use (Palaiodimou et al., 2024). Further, most scores were developed in high-risk populations, such as those with embolic stroke of unknown source, limiting their wide-spread use in the post-stroke setting. Therefore, it was unsurprising that both chosen risk scores, when applied to our unselected patient cohort, showed lower predictive performances than reported in literature (AS5F AUC 0.602, Brown-ESUS AF AUC 0.612). These findings emphasize the need for novel, easily accessible markers that may improve prediction models for occult AF detection.

Therefore, HRV parameters attained through routine 24 h Holter ECG recordings are appealing and as presented through our study, deserve clinical application. Three parameters, namely PNN50, rMSSD, and SDSD, demonstrated higher discriminatory AUC values compared to the AS5F and Brown-ESUS AF score for predicting AF detection upon follow-up within our study cohort (Tables 2, 3). In addition, hierarchical logistic regression analyses demonstrated that PNN50, rMSSD, and SDSD provided significant additive predictive value beyond both the Brown-ESUS AF and AS5F scores, as evidenced by highly significant Omnibus likelihood ratio tests. Through our assessment, we were able to establish cut-off values for all three of these parameters, which may enhance clinical applicability and ease of use in future validation studies. While PNN50, rMSSD, and SDSD were not siginifcantly associated with AF as continuous variables, ROC analysis demostrated acceptable discrimation, and cut-offs derived by the Youden index revealed robust assocations in dichotomized analyses. Therefore these findings should be considered exploratory and wararant external validation.

Our study is not first to report association of individual HRV parameters with incident AF. In the population-based Rotterdam study, which included patients ≥55 years of age, higher rMSSD values were associated with incident AF in both men and women (Geurts et al., 2023). In a Korean study, investigating the incidence of AF in hypertensive patients by Kim et al. (2022) PNN50 and rMSSD showed a significant association with AF (Kim et al., 2022). Zhang et al. (2023) demonstrated that higher rMSSD was closely related to recurrent AF after catheter ablation (Zhang et al., 2023). To-date however, no evidence of an association between SDSD and AF detection has been reported in the literature, and no clear cut-offs for HRV time-domain parameters have been established.

Therefore, our study confirms the negative as well as positive predictive value of rMSSD and PNN50, puts forth SDSD as a novel marker of interest, establishes clinical applicability through cut-off related dichotomization and extends the value of all three markers to an unselected stroke and high-risk TIA patient cohorts. Pending future validation, the establishment of dichotomized cut-off values in routinely attained HRV parameters may hold promise in patient selection for prolonged AF monitoring. This is especially true given that nowadays wearable devices, such as smartwatches, that assess HRV parameters automatically offer a practical and viable alternative standard 24 h Holter ECG measurements (Theurl et al., 2023).

### 4.1 Strengths and limitation of the study

Strengths of the study include the uniform recruitment of patients, the real-world setting, the unselected nature of our stroke and TIA cohort without AF as well as the availability of near-complete HRV parameter values for 697 patients in the acute phase. It is important to point out that our cohort differs in two aspects from the cryptogenic/ESUS cohorts traditionally used in occult AF prediction scores. Based on the finding that detection rates of occult AF are similar in patients with presumed cryptogenic (Sanna et al., 2014; Wachter et al., 2013) as well as in small vessel disease or large artery atherosclerotic (Bernstein et al., 2021) cause of stroke, we included all patients with non-AF stroke or non-AF TIA. In addition, we also included patients with high-risk TIA. Furthermore, it is important to point out that the utilization of loop recorders in our cohort was only 5.2%. Still, this reflects current real-world practice, and it has already been established that patients with clinically detected AF carry twice the risk of ischaemic stroke compared those detected through loop-recorders or other devices (Patel and Ruff, 2024). Our 1-year detection rate AF was similar to the 4.9% reported in the AS5F study and to the 3.4% annual post-discharge incidence observed in a previous ischemic stroke cohort (Uphaus et al., 2019; Witsch et al., 2018). Additionally, the Brown-ESUS score was derived from and is recommended for use in a cryptogenic stroke population. Therefore, its application to an entire cohort of all type non-AF stroke and non-AF TIA is not consistent with its intention and potentially relates to its lower prediction capability within our study (Ricci et al., 2018). The exclusion of patients without sinus rhythm may introduce a potential confounding effect in our analysis as our findings may not be fully generalizable to all ischaemic stroke or high-risk TIA patients. The HRV parameters analyzed in this study were derived from automated read-outs of ECG devices used in routine clinical practice, which should be considered a limitation. Furthermore, a limitation of our study is that the HRV data available did not allow investigation of circadian-related changes in autonomic function (Orini et al., 2023; Magnussen et al., 2017). Additionally, we were unable to assess whether comparable predictive results could be obtained from shorter ECG recordings, which may be of clinical relevance for patients unable to undergo prolonged Holter monitoring. Our study cohort included a relatively high proportion of male participants, which may represent a potential limitation or confounder. Male sex is independently associated with AF, and while cumulative risk of AF is generally higher in men across most of the lifespan, lifetime risk becomes similar at older age. Additionally, some evidence suggests that the previously reported protective effect of female sex may be explained by lack of adjustment for height, with female sex potentially associated with higher AF risk after adjustment (Siddiqi et al., 2022).

## 5 Conclusion

In conclusion, HRV parameters, namely PNN50, rMSSD, and SDSD, are associated with AF detection within 1-year post-stroke. These results emphasize on the potential of to-date underused data collected through routine 24 h Holter ECG recordings as well as their potential within novel risk scores of occult AF in stroke patients.

## Data Availability

The raw data supporting the conclusions of this article will be made available by the authors, without undue reservation.
